# Camelina Seed Supplementation at Two Dietary Fat Levels Change Ruminal Bacterial Community Composition in a Dual-Flow Continuous Culture System

**DOI:** 10.3389/fmicb.2017.02147

**Published:** 2017-11-03

**Authors:** Xiaoxia Dai, Paul J. Weimer, Kimberly A. Dill-McFarland, Virginia L. N. Brandao, Garret Suen, Antonio P. Faciola

**Affiliations:** ^1^Department of Animal Sciences, University of Florida, Gainesville, FL, United States; ^2^Department of Bacteriology, University of Wisconsin-Madison, Madison, WI, United States; ^3^Agricultural Research Service, United States Department of Agriculture, Madison, WI, United States

**Keywords:** 16S rRNA sequencing, biohydrogenation, rumen microbiota, PUFA, cellulolytic bacteria

## Abstract

This experiment aimed to determine the effects of camelina seed (CS) supplementation at different dietary fat levels on ruminal bacterial community composition and how it relates to changes in ruminal fermentation in a dual-flow continuous culture system. Diets were randomly assigned to 8 fermenters (1,200–1,250 mL) in a 2 × 2 factorial arrangement of treatments in a replicated 4 × 4 Latin square with four 10-day experimental periods that consisted of 7 days for diet adaptation and 3 days for sample collection. Treatments were: (1) no CS at 5% ether extract (EE, NCS5); (2) no CS at 8% EE (NCS8); (3) 7.7% CS at 5% EE (CS5); and (4) 17.7% CS at 8% EE (CS8). Megalac was used as a control to adjust EE levels. Diets contained 55% orchardgrass hay and 45% concentrate, and fermenters were equally fed a total of 72 g/day (DM basis) twice daily. The bacterial community was determined by sequencing the V4 region of the 16S rRNA gene using the Illumina MiSeq platform. Sequencing data were analyzed using mothur and statistical analyses were performed in R and SAS. The most abundant phyla across treatments were the *Bacteroidetes* and *Firmicutes*, accounting for 49 and 39% of the total sequences, respectively. The bacterial community composition in both liquid and solid fractions of the effluent digesta changed with CS supplementation but not by dietary EE. Including CS in the diets decreased the relative abundances of *Ruminococcus* spp., *Fibrobacter* spp., and *Butyrivibrio* spp. The most abundant genus across treatments, *Prevotella*, was reduced by high dietary EE levels, while *Megasphaera* and *Succinivibrio* were increased by CS supplementation in the liquid fraction. Correlatively, the concentration of acetate was decreased while propionate increased; C18:0 was decreased and polyunsaturated fatty acids, especially C18:2 n-6 and C18:3 n-3, were increased by CS supplementation. Based on the correlation analysis between genera and fermentation end products, this study revealed that CS supplementation could be energetically beneficial to dairy cows by increasing propionate-producing bacteria and suppressing ruminal bacteria associated with biohydrogenation. However, attention should be given to avoid the effects of CS supplementation on suppressing cellulolytic bacteria.

## Introduction

Ruminal microorganisms are important for health and performance of the host (Russell, 2002), and they are estimated to supply around 80% of the energy and 50% of the protein required by the host animal (Doi and Kosugi, 2004); therefore, changes in the ruminal bacteria community composition (BCC) could affect animal production (Jewell et al., 2015). In ruminants, ruminal BCC is highly responsive to changes on the physical, chemical, and predatory environment created by protozoal in the rumen, as well as by genetics of the host animals (Benson et al., 2010; Weimer et al., 2010a). Different dietary regimes are the main causes for changes in the ruminal environment and thus affecting ruminal BCC (Dehority and Orpin, 1997; Mohammed et al., 2014).

Dietary fat sources, especially those rich in polyunsaturated fatty acids (PUFA), have been reported to decrease ruminal biohydrogenation (BH) by decreasing the abundance of bacteria in the genera *Butyrivibrio* and *Pseudobutyrivibrio* as well as the species *Clostridium proteoclasticum* (Polan et al., 1964; Paillard et al., 2007; Boeckaert et al., 2008). Ruminal cellulolytic species such as *Fibrobacter succinogenes, Ruminococcus flavefaciens*, and *Ruminococcus albus* were also showed to decrease by high amount of PUFA in the diet (Maczulak et al., 1981; Maia et al., 2007). Importantly, decrease BH in the rumen will increase the content of desirable fatty acids (FA) in milk and meat, which may have human health implications and commercial applications. Camelina, a member of the mustard family, is easily adapted to different climate and soil types. Camelina seed (CS) is rich in PUFA (around 73.9% of total FA), especially linolenic acid (Hurtaud and Peyraud, 2007), indicating that CS could be used as a high-quality lipid supplement for ruminants. However, glucosinolates and erucic acid present in CS could impair animal health (Kramer et al., 1990) and affect animal performance.

Dietary supplements of CS or camelina oil have been shown to increase the amounts of *cis*-9 18:1, *cis*-9, *trans*-11 CLA, and 18:3 n-3 FA in the milk of lactating cows (Halmemies-Beauchet-Filleau et al., 2011). However, to our knowledge, the effects of CS supplementation on ruminal BCC and its relationship to ruminal fermentation and metabolite production have not been sufficiently studied. Therefore, the objective of this study was to determine the effects of CS supplementation at two dietary ether extract (EE) levels on ruminal BCC, and further understand how it relates to changes in ruminal fermentation and metabolite production. To accomplish this, we utilized a dual-flow continuous culture system. We hypothesized that the ruminal BCC would change by CS supplementation as well as by the two dietary EE levels, resulting in alterations in ruminal fermentation and metabolite production.

## Materials and Methods

Care and handling of ruminal fluid donor animals were conducted according to a protocol approved by the University of Nevada, Reno, Institutional Animal Care and Use Committee (protocol number 00588).

### Experimental Design and Diets

Diets were randomly assigned to eight 1,200 – 1,250 mL dual-flow continuous culture fermenters (Omni-Culture Plus; Virtis Co. Inc., Gardiner, NY, United States) similar to that originally described by (Hoover et al., 1976), and recently modified by Del Bianco Benedeti et al. (2015), Silva et al. (2016) and Paula et al. (2017). Treatments were arranged in a 2 × 2 factorial design (with or without CS at 5 or 8% total dietary EE) in a replicate 4 × 4 Latin square design with four 10-days experimental periods, consisting of 7 days for diet adaptation and 3 days for sample collections.

Dietary treatments were formulated to meet (National Research Council [NRC], 2001) recommendations for a lactating dairy cow of 650 kg BW producing 35 kg of milk per day. Diets contained 55% orchardgrass hay and 45% concentrate (DM basis; Brandao et al., under review). Treatments were: (1) no CS at 5% EE (NCS5); (2) no CS at 8% EE (NCS8); (3) 7.7% CS at 5% EE (CS5); and (4) 17.7% CS at 8% EE (CS8). Differences in EE levels were established by adjusting the concentrations of Megalac (Church & Dwight Co. Inc., Princeton, NJ, United States), which was used as a control given its little effect on ruminal BCC and fermentation (Grummer, 1988; Chouinard et al., 1998; Theurer et al., 2009); diets were formulated to be isonitrogenous. Dietary ingredients and chemical compositions are present in Supplementary Table 1. Dietary ingredients were ground to pass a 2 mm screen (Wiley mill; Thomson Scientific., Philadelphia, PA, United States) and ground orchardgrass hay was pelleted. Diets were weighed and stored at room temperature in a labeled and sealed plastic bag.

### Dual-Flow Culture System Operation

Ruminal fluid was collected 2 h after morning feeding from 2 ruminally cannulated steers (average BW of 910.5 ± 34.5 kg) fed a 55:45 forage: concentrate diet. The ruminal digesta was collected from the ventral, central, and dorsal areas of the rumen and then strained through four layers of cheesecloth, and a total 12 L of ruminal fluid was filtered into pre-warmed thermal containers. Ruminal fluid was homogenized, infused with N_2_ to maintain the anaerobic environment, and kept at 39°C in a pre-heated water bath.

About 1,250 mL of ruminal fluid was poured into each fermentation vessel until it cleared the overflow spout. During the whole experiment, fermenters were maintained at 39°C and N_2_ (40 mL/min) was continuously infused to maintain anaerobic conditions. The fermenter content was continuously agitated by a central propeller apparatus driven by magnets at a rate of 155 rpm. Artificial saliva (Weller and Pilgrim, 1974) containing 0.4 g/L of urea to simulate recycled N was continuously infused at 2.2 mL/min into vessels. Saliva and liquid flow were measured twice daily for consistency. The solid dilution rate (5.5%) and liquid dilution rate (11.0%) were maintained constantly by regulating buffer input and solid and liquid removal, to mimic *in vivo* passage rates. Each fermenter was manually fed 72 g/d (DM basis) of diet divided into two equal portions at 0800 and 2000 h.

### Microbial Sample Collections and Processing

On day 5, digesta effluent containers were submerged approximately two-thirds of the way in a chilled (2°C) water bath to prevent further microbial fermentation. On days 7, 8, and 9, liquid (15 mL) was collected from liquid effluent container of each fermenter into a 50-mL centrifuge tube at 2, 6, and 10 h after morning feeding and combined into a sample of 45 mL per fermenter per day. At the same time points, solid particles (100, 200, and 200 mL) were collected from solid effluent containers of each fermenter and strained through 4-layer of cheesecloth. A total of 25 g solid particles were combined into a 50 mL Corning centrifuge tube per fermenter per day. Liquid and solid samples were stored at -80°C for further DNA extraction.

### Genomic DNA Extraction

On the day of DNA extraction, liquid and solid samples were thawed and 15 mL of liquid and 8.33 g of solid from each day of the three collection days were combined separately, to yield a total of 45 mL of liquid sample and 25 g solid sample per fermenter per period. Total genomic DNA was extracted separately from solid and liquid samples following the methods described by Stevenson and Weimer (2007). This method is similar to the Phenol-chloroform with bead beating II (PSCA) method (Henderson et al., 2013), which provided the greatest yield of DNA from ruminal samples of 13 methods tested, and is thus likely to yield DNA representative of the ruminal BCC. Briefly, solid samples were blended with DNA extraction buffer to dislodge particle adherent cells and centrifuged at a low speed to remove large particles. For all samples, bacterial cells were collected by high-speed centrifugation. Cells were lysed by bead-beating with zirconia/silica beads (BioSpec Products, Bartlesville, OK, United States) on a Mini-Beadbeater (Biospec Products) and heating to 60°C with 20% sodium dodecyl sulfate and phenol. DNA was purified by repeated phenol and phenol-chloroform extraction. DNA was isopropanol precipitated with Na acetate and then resuspended in Tris-EDTA (TE) buffer. After quantification by Qubit^®^ Fluorometer (Invitrogen, San Diego, CA, United States), DNA samples were stored at -80°C.

### Volatile Fatty Acids Analysis

Total volatile fatty acids (VFA), acetate, propionate, butyrate, valerate, isobutyrate and isovalerate were analyzed as described by Brandao et al. (under review). Lactate, formate and succinate were analyzed following the method of Weimer et al. (1991) and analyzed by high performance liquid chromatograph (HPLC). The column used for analysis was a Bio-Rad HPX-87H, 300 mm length with 4.6 mm i.d (Bio-Rad, Bio-Rad Laboratories, Inn, CA, United States) with a flow rate of 0.7 mL/min. The VFA components were identified by comparison of retention time with a mixture of fermentation product standards (10 mM each); previous studies have shown that the refractive index detection used for this assay provides a linear response with VFA concentration up to at least 300 mM (Dill-McFarland et al., 2014).

### DNA Amplification, Library Preparation and Sequencing

The variable 4 (V4) region of the bacterial 16S rRNA gene was amplified following Kozich et al. (2013). A total of 20 ng DNA, 0.4 μM each primer, and 12.5 μL 2X KAPA Hotstart ReadyMix (Kapa Biosystems, Wilmington, MA, United States), and water to 25-μL were used for PCR reaction by Bio-Rad C1000 Touch^TM^ Thermal Cycler (BIO-RAD, Hercules, CA, United States). Amplification conditions were as follows: initial denaturation of 95°C for 3 min, 25 cycles of 95°C for 30 s, 55°C for 30 s and 72°C for 30 s, and the final elongation at 72°C for 5 min. Removal of primer and small DNA fragment contaminants were performed by gel extraction using a 1% low melt agarose gel (National Diagnostics, Atlanta, GA, United States) visualized using SYBR^TM^ Safe DNA gel stain (Invitrogen, San Diego, CA, United States). The amplicons were then extracted using Zymo Gel DNA Recovery Kit (Zymo Research, Irvine, CA, United States). The concentrations of purified PCR products were quantified by Qubit^®^ Fluorometer (Invitrogen, San Diego, CA, United States) and equimolar pooled to 4 nM. Libraries plus 10% PhiX control DNA were sequenced on an Illumina Miseq using MiSeq^®^ reagent kit V3 (2 × 250 cycles, Illumina, San Diego, CA, United States), according to the manufacturer’s protocol. All sequencing data have been submitted to Sequence Read Archive (SRA), accession number SRP115834.

### Data Analysis

Sequence processing and data analysis were performed using mothur v.1.38.1 following the MiSeq SOP (Schloss et al., 2009; Kozich et al., 2013) unless specified (Supplementary Text 1). Briefly, sequences were trimmed and filtered based on quality. The unique sequences were aligned against the SILVA 16S rRNA gene reference alignment database (Pruesse et al., 2007). Sequences that were two or fewer base pairs different were considered the same and grouped by *pre.cluster* (diffs = 2). Chimeras were detected and removed by *chimera.uchime* and *remove.seqs*. Sequences were clustered into 97% operational taxonomic units (OTUs) using the average neighbor algorithm. Classification of OTUs were performed based on the GreenGenes database (DeSantis et al., 2006), August 2013 release, with a bootstrap cutoff of 80. Sequences classified as *Eukaryota, Archaea, Cyanobacteria* or unknown were removed from the all subsequent analyses. Although Cyanobacteria have not been identified in ruminal samples, they have been identified in hindgut communities (Di Rienzi et al., 2013; Soo et al., 2014) so it is possible that we have excluded them in the interest of not confounding them with chloroplasts. All samples were normalized by subsampling to 13,000 sequences, the size of the smallest sample. Alpha richness (Chao and ACE), diversity (Shannon, inverse Simpson), OTU counts, and coverage metrics were obtained by using *summary.single* from normalized data.

Different OTUs belonging to the same phyla, families, and genera in both liquid and solid phases were summarized in Python v 3.6.0, based on OTU count and taxonomy files generated using mothur. The relative abundance of different phyla, families, and genera were calculated as the represented sequence reads divided by the total sequences. Only the average relative abundances of phyla, families and genera higher than 0.1% across all samples were considered further.

The 100 most abundance OTUs were determined based on the average relative abundance across all samples. The relative abundances of OTUs that were significantly affected by treatment were first log-transformed and then heatmaps were generated using Genesis 1.8.1 (Sturn et al., 2002). The expression value among different groups was normalized using the built-in function in Genesis and the corresponding phylogenetic tree was generated in MEGA7 (Kumar et al., 2016) based on representative sequences obtained from mothur.

### Statistical Analyses

Parts of the statistical analyses were carried out in R [vegan package, (Oksanen et al., 2015)]. Total microbial community structure (Bray-Curtis) and composition (Jaccard) were calculated from normalized OTU data and visualized by non-metric multidimensional scaling (NMDS) plots. The PERMANOVA was run to determine the differences in community structure and composition between the phases (liquid or solid), CS treatment (with and without), and dietary EE level (low and high) by using the *adonis* function in *vegan*, with the Benjamini–Hochberg correction for multiple comparisons when necessary.

Factorial effects — CS supplementation, dietary EE levels, and the interactions of the two — on the community diversity and richness in both of liquid and solid phase were assessed using the MIXED procedure of SAS 9.4 software (Cary, NC, United States). The factorial effects on the relative abundances of the top 100 OTUs as well as families and genera higher than 0.1% were further analyzed by the MIXED procedure in SAS. Correlations between bacterial taxa and the concentrations of VFA and long-chain fatty acids were determined using the Pearson CORR procedure in SAS, and *P*-values were corrected with false discovery rate. All data were expressed as the least square mean ± SEM and considered significant if *P* ≤ 0.05, with tendencies identified if 0.05 < *P* ≤ 0.10.

## Results

### DNA Sequence Data

One sample from period 2 and one sample from period 4 were excluded due to poor PCR amplification. A total of 3,313,817 high-quality sequences were retained after filtering through mothur. Sequence coverage sufficiently met a Good’s coverage greater than 98.5% for all samples (Supplementary Table 2). Across all the samples in the effluent, 40,088 OTUs were identified. In total, 98% of OTUs were classified at the phyla level, 83% at the family level, and 66% at the genus level (Supplementary Table 3).

A total of 26 phyla were identified within the ruminal bacterial population from all samples. Most samples were dominated by sequences belonging to the *Bacteroidetes* and *Firmicutes* across all the treatments, accounting for a combined total of 88% of the total sequences (49 and 39% of total sequences, respectively). The phylum *Proteobacteria* represented 6.5% and *Tenericutes* represented 1.1% of the total sequences, on average (Supplementary Table 3.1). A total of 123 families were identified and the families *Prevotellaceae, Lachnospiraceae*, and *Erysipelotrichaceae* had the largest relative abundance across all the treatments: 35, 15, and 11% of total sequences, respectively (Supplementary Table 3.2). A total of 196 genera were identified, with *Prevotella* as the predominant genus, representing 35% of the relative abundance across all treatments. The genera *Succinivibrio, Butyrivibrio, Pseudobutyrivibrio*, and *Megasphaera* represented 4.1, 3.9, 2.7, and 1.9% of the total sequences across all the treatments, respectively (Supplementary Table 3.3).

### Effects of CS Supplementation and Two EE Levels on Bacterial Community Compositions

**FIGURE 1:**
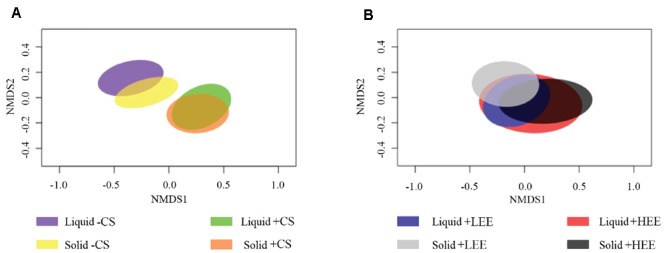
Non-metric multidimensional scaling (NMDS) plots of the Bray-Curtis similarity comparing the ruminal bacterial community composition of **(A)** effect of camelina seed supplementation, and **(B)** effect of different dietary ether extract levels (EE) [8% EE (HEE) vs. 5% EE (LEE)]. Ellipses represent 95% confidence intervals.

The liquid-associated and solid-associated ruminal BCC were significantly different in both composition and structure (composition and abundance of ruminal BCC) (Supplementary Figure 1 and Table 4). The NMDS plots showed that ruminal BCC with CS supplementation were clearly separated from ruminal BCC without CS supplementation in both liquid and solid fractions (*P* < 0.01, **Figure 1A**), indicating that ruminal BCC was altered by CS supplementation. However, the dietary EE levels did not significantly change ruminal BCC in either liquid and solid fractions (**Figure 1B**).

**FIGURE 2:**
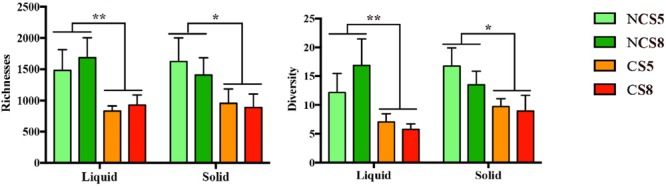
Effect of camelina seed (CS) supplementation and dietary EE levels on the richness and diversity of ruminal bacterial community composition in the liquid and solid fractions. Richness was calculated by the Chao-index and diversity by the inverse-Simpson index. NCS5, non-camelina seed inclusion at 5% dietary EE; NCS8, non-camelina seed inclusion at 8% dietary EE; CS5, low camelina seed inclusion at 5% dietary EE; CS8, high camelina seed inclusion at 8% dietary EE. ^∗∗^*P* < 0.01; ^∗^*P* < 0.05.

Dietary CS supplementation decreased the richness (*P* < 0.01) and diversity (*P* = 0.01) of ruminal BCC in both liquid and solid fractions (**Figure 2**). However, dietary EE levels had no effect on the richness or diversity of ruminal BCC. There was no interaction of CS and dietary EE levels on the richness and diversity of ruminal BCC.

**FIGURE 3:**
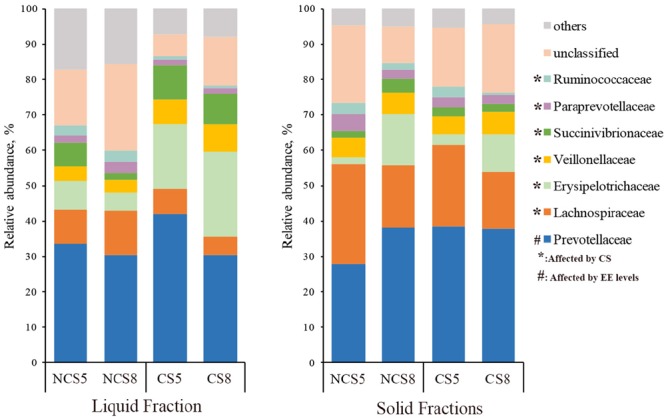
Effects of camelina seed supplementation and dietary EE levels on the relative abundance of bacterial families in the ruminal liquid and solid fractions. NCS5, non-camelina seed inclusion at 5% dietary EE; NCS8, non-camelina seed inclusion at 8% dietary EE; CS5, low camelina seed inclusion at 5% dietary EE; CS8, high camelina seed inclusion at 8% dietary EE.

Effects of CS supplementation and dietary EE levels on the relative abundance of families are presented in Supplementary Table 5. Dietary CS supplementation decreased the relative abundance of *Lachnospiraceae* (*P* < 0.01), *Ruminococcaceae* (*P* < 0.05), *Paraprevotellaceae* (*P* < 0.05) and *Fibrobacteraceae* (*P* < 0.05), but increased the relative abundance of *Erysipelotrichaceae* (*P* < 0.01) in both liquid and solid fractions (**Figure 3**). Dietary CS supplementation increased the relative abundance of *Succinivibrionaceae* (*P* = 0.01) and *Veillonellaceae* (*P* < 0.01) in the liquid fraction. Dietary EE at 8% decreased the relative abundance of *Prevotellaceae* (*P* = 0.02) in the liquid fraction.

**FIGURE 4:**
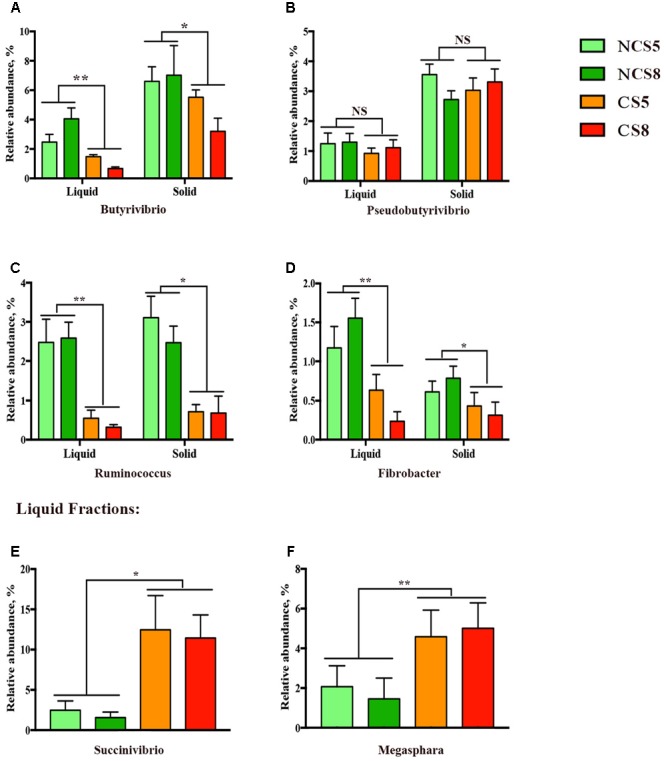
Effects of camelina seed supplementation and dietary EE levels on the relative abundance of bacterial genera (**A** = *Butyrivibrio*; **B** = *Pseudobutyrivibrio*; **C** = *Ruminococcus*; **D** = *Fibrobacter*; **E** = *Succinivibrio*, and **F** = *Megasphara*) in ruminal liquid and solid fractions. NCS5, non-camelina seed inclusion at 5% dietary EE; NCS8, non-camelina seed inclusion at 8% dietary EE; CS5, low camelina seed inclusion at 5% dietary EE; CS8, high camelina seed inclusion at 8% dietary EE. ^∗∗^*P* < 0.01; ^∗^*P* < 0.05; NS, not significant.

Effects of CS supplementation and dietary EE levels on the relative abundance of genera are presented in Supplementary Table 6. Dietary CS supplementation decreased the relative abundance *of Butyrivibrio* (*P* < 0.05, **Figure 4A**), but had no effect on the relative abundance of *Pseudobutyrivibrio* (**Figure 4B**) in both liquid and solid fractions. The relative abundance of *Ruminococcus* (*P* < 0.05, **Figure 4C**) and *Fibrobacter* (*P* < 0.01, **Figure 4D**) was decreased in both liquid and solid fractions when CS was included in the diets. However, CS supplementation increased the relative abundances of *Succinivibrio* (*P* = 0.02, **Figure 4E**) and *Megasphaera* (*P* < 0.01, **Figure 4F**), and also increased the relative abundance of *Clostridium* (*P* < 0.01) in the liquid fraction. The relative abundance of *Prevotella* (*P* = 0.02) was decreased by 8% dietary EE level in the liquid fraction.

**FIGURE 5:**
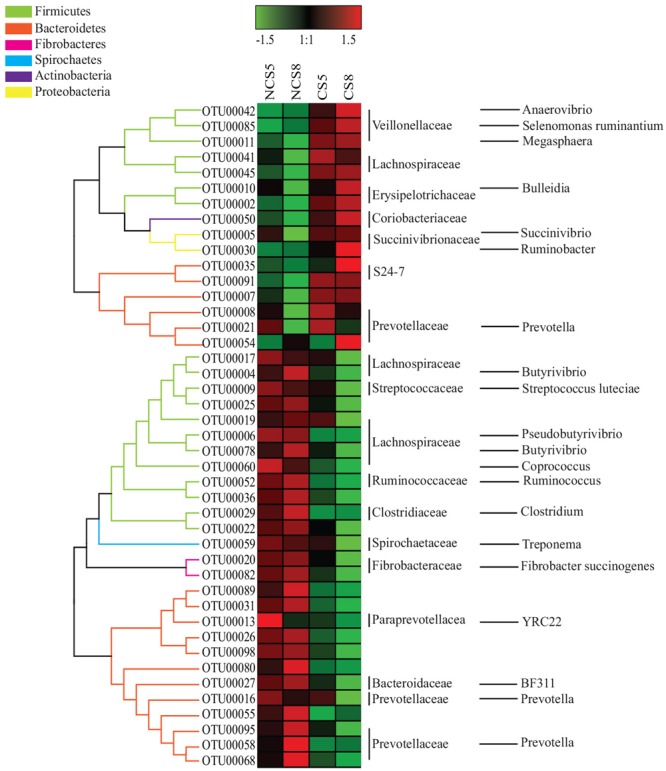
Effects of camelina seed supplementation and dietary EE levels on the relative abundance of OTUs in the liquid fraction. The evolutionary history was inferred by using the Maximum Likelihood method based on the Tamura-Nei model. NCS5, non-camelina seed inclusion at 5% dietary EE; NCS8, non-camelina seed inclusion at 8% dietary EE; CS5, low camelina seed inclusion at 5% dietary EE; CS8, high camelina seed inclusion at 8% dietary EE.

**FIGURE 6:**
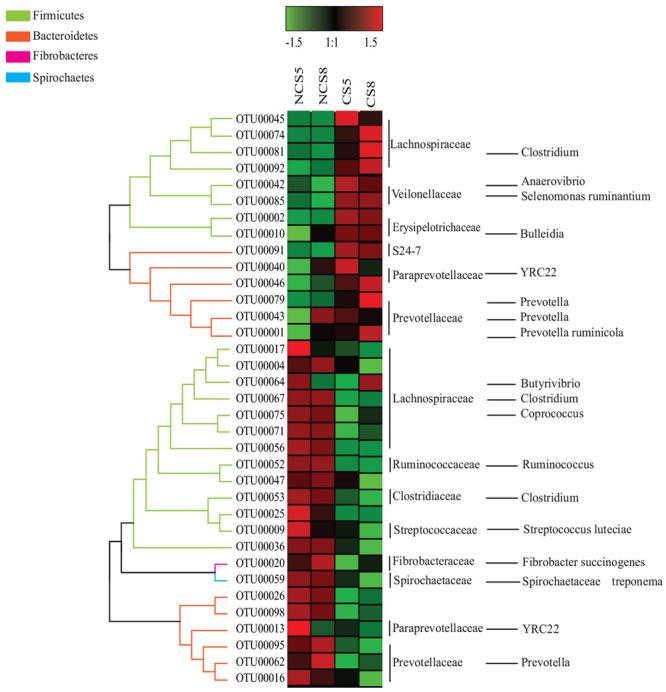
Effects of camelina seed supplementation and dietary EE levels on the relative abundance of OTUs in the solid fraction. The evolutionary history was inferred by using the Maximum Likelihood method based on the Tamura-Nei model. NCS5, non-camelina seed inclusion at 5% dietary EE; NCS8, non-camelina seed inclusion at 8% dietary EE; CS5, low camelina seed inclusion at 5% dietary EE; CS8, high camelina seed inclusion at 8% dietary EE.

Dietary CS supplementation increased the relative abundance of OTUs belonging to families *Veillonellaceae* (*Anaerovibrio, Selenomonas ruminantium*, and *Megasphaera*), *Erysipelotrichaceae* (*Bulleidia*), *Succinivibrionaceae* (*Succinivibrio* and *Ruminobacter*), and S24-7, but decreased the relative abundance of OTUs belonging to families *Ruminococcaceae* (*Ruminococcus*), *Fibrobacteraceae* (*Fibrobacter succinogenes*) and *Lachnospiraceae* (*Butyrivibrio* and *Pseudobutyrivibrio*) in both liquid and solid fractions (**Figures 5, 6**).

### Effects of CS Supplementation and Two EE Levels on the Concentrations of VFA and Long Chain Fatty Acids and Their Correlations with Ruminal BCC

Dietary CS supplementation was showed to increase of propionate, valerate and C_4_ and C_5_-branched chain VFA but decreased the concentration of total VFA and acetate in our companion study (Brandao et al., under review). The concentrations of succinate, lactate, and formate were not affected by treatments (**Table 1**). Dietary EE levels and the interaction of CS and dietary EE levels had no effects on the concentrations of any of the short-chain organic acids.

**Table 1:** Effects of camelina seed supplementation at two dietary ether extract levels on succinate, formate and lactate concentrations (mM) in a dual-flow continuous culture system.

Item	Treatment^1^				SEM	*P-* value^2^		
	NCS5	NCS8	CS5	CS8		CS	EE	CS x EE
Succinate, mM	0.36	0.42	0.93	0.60	0.25	0.16	0.60	0.44
Lactate, mM	2.90	1.81	2.86	3.02	0.84	0.44	0.54	0.41
Formate, mM	0.33	0.02	0.34	0.2	0.13	0.49	0.11	0.55

*^1^NCS5, non-camelina seed inclusion at 5% dietary EE; NCS8, non-camelina seed inclusion at 8% dietary EE; CS5, low camelina seed inclusion at 5% dietary EE; CS8, high camelina seed inclusion at 8% dietary EE. ^2^CS, effects of camelina seed; EE, effects of dietary EE level; CS × EE, the interaction between CS and EE.*

**FIGURE 7:**
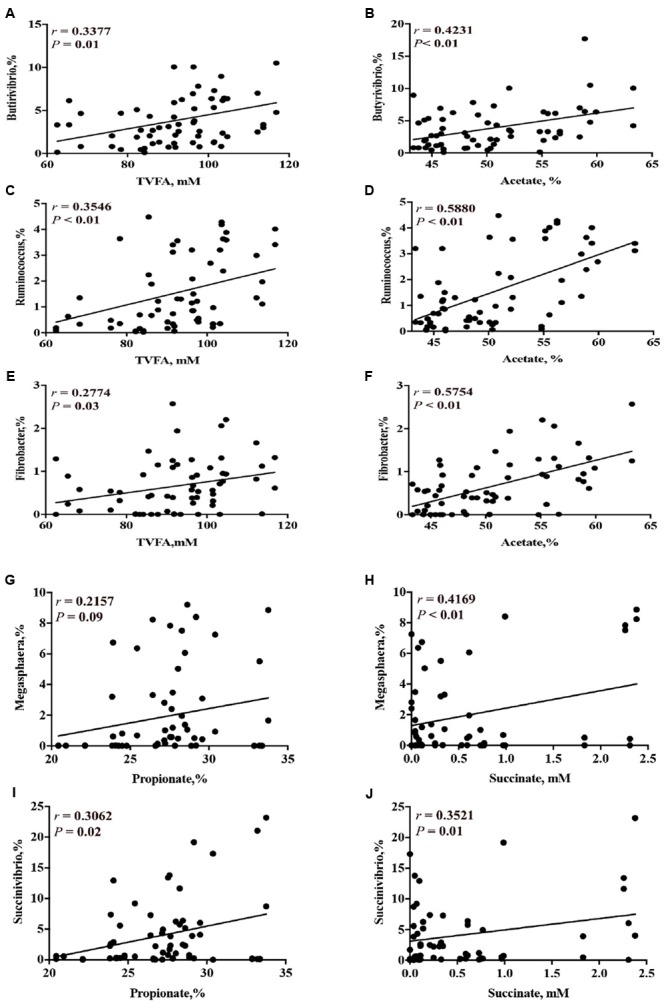
Correlation of concentration of total VFA and the relative abundance of ruminal bacterial genera (**A** = *Butyrivibrio*; **C** = *Ruminococcus*; **E** = *Fibrobacter*); concentration of acetate with the relative abundance of ruminal bacterial genera (**B** = *Butyrivibrio*; **D** = *Ruminococcus*; **F** = *Fibrobacter*); concentration of propionate with the relative abundance of ruminal bacterial genera (**G** = *Megasphaera*; **I** = *Succinivibrio*); and concentration of succinate with the relative abundance of genera (**H** = *Megasphaera*; **J** = *Succinivibrio*). *r* = Pearson’s *r* coefficient.

Correlation analysis results between the concentrations of short-chain organic acids and the relative abundance of ruminal bacterial genera are presented on Supplementary Table 7.1. Based on the correlation analysis, the relative abundance of genera that were decreased by CS supplementation (i.e., *Butyrivibrio, Ruminococcus*, and *Fibrobacter*) had a linear positive correlation with the concentration of total VFA (*P* < 0.05) and acetate (*P* < 0.01) (**Figures 7A–F**). The relative abundance of genera that were increased by CS supplementation (i.e., *Succinivibrio* and *Megasphaera*) had a linear positive correlation with the concentration of propionate and succinate (*P* < 0.01, **Figures 7G–J**).

In our companion study, dietary CS supplementation was decreased the concentrations of C18:0 (*P* < 0.01) and the total saturated fatty acids (*P* < 0.01), but increased the concentrations of C18:2 n-6 (*P* < 0.01), C18:2 tran-10, cis-12 (*P* < 0.01), total unsaturated FA (*P* < 0.01), Monounsaturated FA and PUFA (*P* < 0.01). The concentration of C18:3 n-3 and erucic acid (C22:1*n-*9) were much higher in the 8% dietary EE level when CS was included in the diets (Brandao et al., under review).

**FIGURE 8:**
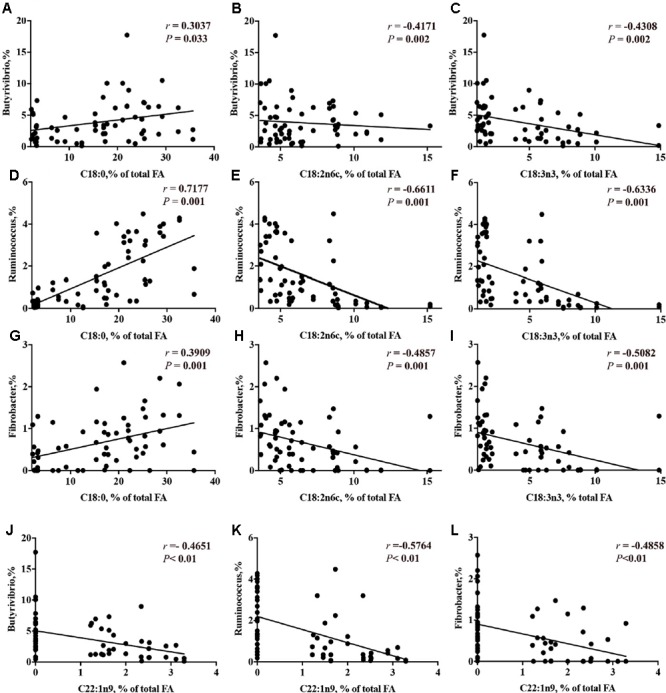
Correlation of concentrations of C18:0 with the relative abundance of ruminal bacterial genera (**A** = *Butyrivibrio*; **D** = *Ruminococcus*; **G** = *Fibrobacter*); concentration of C18:2n6c with the relative abundance of ruminal bacterial genera (**B** = *Butyrivibrio*; **E** = *Ruminococcus*; **H** = *Fibrobacter*); concentration of C18:3n3 with the relative abundance of ruminal bacterial genera (**C** = *Butyrivibrio*; **F** = *Ruminococcus*; **I** = *Fibrobacter*); and concentration of C22:1 n-9 with the relative abundance of ruminal bacterial genera (**J** = *Butyrivibrio*; **K** = *Ruminococcus*; **L** = *Fibrobacter*). *r* = Pearson’s *r* coefficient.

The correlation analysis results between the concentration of long chain FA and the relative abundance of genera are presented in Supplementary Table 7.2. The relative abundance of genera that were decreased by CS supplementation (i.e., *Butyrivibrio, Ruminococcus* and *Fibrobacter*) had a linear positive correlation with C18:0 (*P* < 0.05) and a linear negative correlation with the concentrations of C18:2 n-6, C18:3 n-3 (*P* < 0.01, **Figures 8A–I**). The relative abundance of *Megasphaera* had no correlation with the concentration of *trans*-10, *cis*-12 C18:2. The concentration of erucic acid (22:1*n-*9) had a linear negative correlation with the genera *Butyrivibrio* (-46.5%, **Figure 8J**), *Ruminococcus* (-57.6%, **Figure 8K**) and *Fibrobacter* (-48.6%, **Figure 8L**).

## Discussion

In the present study, ruminal bacterial populations are distinguished between solid and liquid fraction when treated by CS supplementation at two dietary EE levels. This is important given that ruminal bacterial populations involved in lipolysis or BH can be divided into liquid- or solid-associated bacteria (Czerkawski, 1986; Legay-Carmier and Bauchart, 1989). Dietary CS altered ruminal BCC and decreased the richness and diversity of bacteria in both liquid and solid fractions when compared with Megalac supplementation in the present study, which supports our hypothesis. The Megalac added in the diet was considered a control due to its higher concentration of saturated FA and expected less deleterious effects on ruminal environment (Grummer, 1988; Chouinard et al., 1998; Theurer et al., 2009). This may reflect PUFA toxicity to ruminal bacteria, which is particularly the case for cellulolytic species and butyrate producers (Maia et al., 2007). In the present study, dietary CS decreased the relative abundance of cellulolytic bacteria (*Ruminococcus* spp. and *Fibrobacter* spp.) in both liquid and solid fractions. This is consistent with the report of Maia et al. (2007) that cellulolytic bacteria did not grow in the presence of PUFA at 50 μg/ml, especially in the presence of α-linolenic acid (ALA). Ebrahimi et al. (2017) also found inhibition of *Ruminococcus albus* and *Ruminococcus flavefaciens* with increased dietary ALA.

The proportions of acetate, propionate, and butyrate generated in the rumen are affected by the species and quantities of ruminal bacteria (Stewart and Flint, 1992; Tajima et al., 2000). As the VFA are the main end products of ruminal fermentation of ruminal bacteria (Russell, 2002). In the present study, CS supplementation increased the molar proportion of propionate, whereas it decreased the molar proportion of acetate, which was consistent with our companion study in which ruminal NDF and ADF digestibilities were significantly decreased by CS supplementation (Brandao et al., under review). CS supplementation altered ruminal fermentation, possibly by selecting for organisms that produce more propionate and against those that produce more acetate. The decrease of acetate by CS supplementation could be due to the toxic effect of ALA and LA on *Ruminococci* spp. and *Butyrivibrio* spp., which are important producers of acetate and butyrate (Bergman, 1990), and their relative abundance displayed linear positive correlation with acetate in the present study.

The increase of propionate by dietary CS supplementation may be related to the increase of *Selenomonas. ruminantium* and *Megasphaera elsdenii* when CS was supplemented in diets in the present study. *S. ruminantium* is the specie that utilizes the succinate-propionate pathway to transform lactate into propionate. Nisbet and Martin (1991) reported that with an increasing amount of *S. ruminantium*, a decrease in the acetate: propionate ratio was observed *in vitro*, which was also found in the present study. Even though *M. elsdenii* is the main lactate-utilizing species in the rumen (Russell, 2002), Counotte et al. (1981) found that more than 70% of lactate was fermented by *M. elsdenii* via the acrylate pathway in dairy cows and that propionate was the final product. Henning et al. (2010) reported that high-producing dairy cows treated with *M. elsdenii* after calving showed significantly increased in propionate in the rumen. Therefore, increase of propionate upon CS supplementation in the present study could be due to the increase of relative abundance of *Selenomonas* spp. and *Megasphaera* spp., which is consistent with a tendency of linear correlation between the relative abundance of *Megasphaera* spp. and *Selenomonas* spp with propionate concentration. The abundance of these two taxa were also positively correlated with valerate concentration, which is in agreement with the fact that *M. elsdenii* is one of the few bacterial species known to produce valerate from non-protein sources (Marounek et al., 1989).

The CS used in the present study contained approximately 55% PUFA of total FA. Ingested PUFA may be converted into saturated FA due to bacterial BH in the rumen (Kim et al., 2008). The pathway of BH in the rumen can be simplified as the conversion of free C18 PUFA to conjugated dienoic or trienoic acids, then to C18:1 and finally through a reduction mechanism to C18:0 (Kemp and Lander, 1984). Bacteria involved in BH have been classified as Group A and Group B. Group A bacteria hydrogenate PUFA 18:2n-6 and 18:3n-3 to *trans*-11 18:1; Group B bacteria hydrogenate the same FA to C18:0 (Harfoot and Hazlewood, 1997). Phylogenetic analysis of recent isolates has shown that C18:0-forming bacteria, like the most active Group A bacteria, are part of the *Butyrivibrio* group, including the genera *Butyrivibrio* and *Pseudobutyrivibrio* (Kopečný et al., 2003; Paillard et al., 2007; Boeckaert et al., 2008). In the present study, the relative abundance of *Butyrivibrio* was decreased when CS was supplemented; however, no effect was found on *Pseudobutyrivibrio. Butyrivibrio (Butyrivibrio hungatei*), that have been observed to not grow in the presence of PUFA, especially in the presence of ALA (Maia et al., 2007), while no effect on the relative abundance of *Pseudobutyrivibrio* was observed. This may be because *Pseudobutyrivibrio* is more resistant to ALA. The relative abundance of *Butyrivibrio* had significant negative correlations with C18:2 n-6 and C18:3 n-3. Therefore, the increase in the concentrations of C18:2 n-6 and C18:3 n-3 may result from the reduction of *Butyrivibrio* group by CS supplementation, consistent with the role of *Butyrivibrio* as 18:0-forming bacteria, that is hydrogenating the C18:2n-6 and C18:3n-3 to *trans*-11 C18:1, and ultimately C18:0. The inhibition of *Ruminococcus* spp. by CS supplementation may be also responsible for the reduction of C18:0 and increase of PUFA, as observed by the linear positive correlation with C18:0 and a linear negative correlation with the concentrations of C18:2 n-6, C18:3 n-3.

*Butyrivibrio proteoclasticus* (formerly *Clostridium proteoclastium)* is the only known cultured ruminal species belonging to Group B bacteria (C18:0 producer) converting C18:2 and C18:3 to C18:0, and are among the most sensitive ruminal species to the toxic effects of PUFA (Vossenberg and Joblin, 2003; Wallace et al., 2006; Moon et al., 2008). The concentration of C18:0 was significantly decreased by dietary CS, whereas no *C. proteoclastium* was detected from the present study. Toral et al. (2012) also did not detect any *B. proteoclasticus* when sunflower oil or marine algae were fed to sheep. Therefore, these bacteria may not play a major role in C 18:0 formation in the rumen (Boeckaert et al., 2008), other unclassified *species* may be involved in C18:0 production, as has been suggested by Kim et al. (2008).

Several studies have found that the *Megasphaera* spp. were the major difference in ruminal BCC between milk fat depressed cows and no milk fat depression cows (Weimer et al., 2010b, Mohammed et al., 2012). The relative abundance of *M. elsdenii*. comprised up to 4% of the 16S rRNA gene copy number of the total BCC in MFD cows, but did not exceed 0.02% in cows before onset of milk fat depression (Weimer et al., 2010b). In the present study, we found that *Megasphaera* spp. were increased by CS supplementation in the diets (around 5% of total sequences), which is similar to the study to Weimer et al. (2010b). The concentration of *trans*-10, *cis*-12 CLA was increased by CS supplementation. *Trans*-10, *cis*-12 CLA is associated with modulating fat deposition (Park et al., 1999) and milk fat depression (Shingfield and Griinari, 2007). *M. elsdenii* has been reported to convert linoleic acid (C18:2 n-6) to *trans*-10, *cis-*12 CLA (Kim et al., 2002). However, no significant correlation was found between *Megasphaera* spp. and *trans*-10, *cis*-12 CLA in the present study, suggesting that *Megasphaera* spp. may not be involved in *trans*-10, *cis*-12 CLA production, which was also consistent with the study of Maia et al. (2007). However, direct correlations between *trans*-10, *cis*-12 CLA may be misleading because this acid is an intermediate in the BH pathway, and its measured concentrations may be influenced by the abundance and activity of bacterial species capable of further metabolism of this acid.

Erucic acid (C22:1 n-9) is one of the most abundant of anti-nutrient containing in CS. Erucic acid present in salads (*Diplotaxis* and *Eruca* genera of *Brassicaceae* family) has been reported to have direct antimicrobial effects on both Gram-positive (*Staphylococcus aureus, Staphylococcus epidermidis, Bacillus subtilis*) bacteria and Gram-negative (*Escherichia coli, Pseudomoms aeruginosa, Shigella flexneri, Salmonella typhi, Klebsiella pneumonia*) bacteria in humans (Cavaiuolo and Ferrante, 2014). The concentration of erucic acid was increased by CS supplementation in the present study. Erucic acid also was showed to have negative correlations with *Butyrivibrio* spp., *Ruminococcu* spp. and *Fibrobacter* spp. Therefore, accumulation of erucic after CS supplementation may be toxic to cellulolytic and bacteria involved in BH as well. However, further studies are required to elucidate the effects of erucic acid on ruminal bacteria.

From this study, we also found that the relative abundance of the family *Erysipelotrichaceae* was increased by CS supplementation. This may be due to the high PUFA contents in CS, high concentration of glucosinolates (23.0 mmol/kg, Brandao et al., under review), or erucic acid containing in the CS. However, physiological information of this family is limited due to lack of pure culture representatives for study.

Ruminal BCC richness and diversity were not different between 5 and 8% dietary EE levels in the present study. This indicates that a 3% difference in dietary EE may not be enough to cause differences in ruminal BCC in an *in vitro* system. According to the National Research Council [NRC] (2001), total dietary fat should not exceed 6–7% of dietary DM for lactating cows, therefore, it was expected that the ruminal BCC would be different between 5 and 8% dietary EE. This may be due to lower protozoa numbers *in vitro* (Slyter and Putnam, 1967; Mansfield et al., 1995) or partly due to the fact that certain bacterial species seem to be preferentially enriched under *in vitro* conditions (Weimer et al., 2011). However, bacteria present in continuous culture system may be species that were not easily affected by dietary EE levels. Therefore, further studies *in vivo* may be required to test the difference of dietary EE levels on ruminal BCC.

Even though no difference were found in the ruminal BCC by the two dietary EE levels, we found that the relative abundance of *Prevotellaceae* (*Prevotella*) was decreased by 8% dietary EE level in liquid fraction. The reason for that could be that *Prevotella* is a genus of Gram-negative bacteria and it has a thinner peptidoglycan and porins traverse the outer molecular that act as channels for low molecular weight compounds (Russell, 2002). Therefore, *Prevotella* species are more sensitive to dietary fat supplementation and were reduced by greater dietary EE level (8% EE). *Prevotella* species seem to play an important role in ruminal protein catabolism (Russell, 2002), and their decreased relative abundance at 8% EE is consistent with an observed decrease the flows of NH_3_-N (g/d), non-ammonia nitrogen, bacterial-N and bacterial efficiency in a companion study (Brandao et al., under review).

## Conclusion

Ruminal BCC was affected by CS supplementation but not by dietary EE levels in both liquid and solid fractions. Inclusion of CS in the diets decreased cellulolytic bacteria and altered the ruminal BH process by decreasing the relative abundance of *Butyrivibrio* spp. and increasing *Megasphaera* spp. and *Succinivibrio* spp. Additionally, the concentration of acetate was decreased while propionate was increased; C18:0 was decreased, and PUFA, especially C18:2 n-6 and C18:3 n-3 were increased by CS supplementation. Thus, dietary CS supplementation could be energetically beneficial to dairy cows by increasing the propionate-producing bacteria and useful at suppressing ruminal bacteria associated with BH; however, attention should be given to avoid effects of CS supplementation on suppressing cellulolytic bacteria.

## Author Contributions

Project acquisition: XD, AF, GS, and PW. Trial and project design: XD, VB, and AF. Trial implementation and sample collection: XD and VB. Sample analysis (DNA extraction, PCR, MiSeq): XD, PW, and KD-M. Data Analysis (Statistics and Graphics): XD, KD-M, and PW. Data interpretations: XD, KD-M, and PW. Writing of manuscript: XD. Revision of manuscripts: XD, PW, KD-M, VB, GS, and AF.

## Conflict of Interest Statement

The authors declare that the research was conducted in the absence of any commercial or financial relationships that could be construed as a potential conflict of interest. The reviewer RA and author PW declared their shared affiliation.
