# Supplementary material for: Camelina Seed Supplementation at Two Dietary Fat Levels Change Ruminal Bacterial Community Composition in a Dual-Flow Continuous Culture System
Source: Front Microbiol. 2017 Nov 3;8:2147. doi: 10.3389/fmicb.2017.02147 (PMC5675879; doi:10.3389/fmicb.2017.02147)
Supplement: Supplementary file 3 [file Data_Sheet_1.docx]

Supplementary Material

**Camelina seed supplementation at two dietary fat levels change ruminal bacterial community composition in a dual-flow continuous culture system**

Xiaoxia. Dai, Paul. J. Weimer, Kimberly. A. Dill-McFarland, Virginia. L. N. Brandao, Garret. Suen, and Antonio. P. Faciola

*** Correspondence:** Antonio. P. Faciola: [afaciola@ufl.edu](mailto:afaciola@ufl.edu)

# Supplementary Figures and Tables

## Supplementary Figures


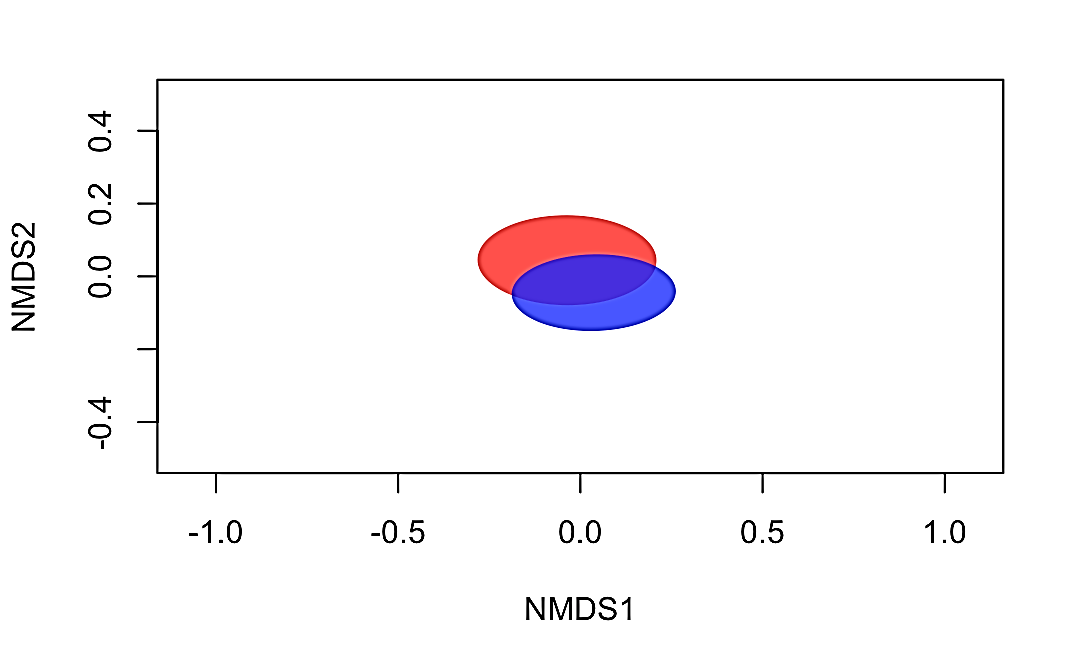


Supplementary Figure 1. Non-Metric multidimensional scaling (NMDS) plot of the Bray-Curtis similarity index compared the liquid-associated bacterial communities (red) and solid-associated bacterial communities (blue). Ellipses represent 95% confidence intervals.

## Supplementary Tables

**Table S1**. Ingredient and chemical composition of the experimental diets

| Item | Diets^1^ | | | |
| --- | --- | --- | --- | --- |
|  | NCS5 | NCS8 | CS5 | CS8 |
| % DM, unless otherwise stated |  |  |  |  |
| Orchardgrass hay | 55.0 | 55.0 | 55.0 | 55.0 |
| Ground corn | 17.5 | 12.4 | 19.2 | 16.3 |
| Canola meal | 22.9 | 23.9 | 16.8 | 9.8 |
| Camelina seed | - | - | 7.7 | 17.7 |
| Megalac^2^ | 3.25 | 7.48 | - | - |
| Minerals^3^ | 1.25 | 1.24 | 1.25 | 1.25 |
| Chemical Composition |  |  |  |  |
| DM, % | 91.9 | 92.4 | 91.8 | 92.3 |
| OM | 92.3 | 91.4 | 93.0 | 93.1 |
| CP | 16.0 | 16.0 | 16.0 | 16.0 |
| NDF | 35.5 | 35.3 | 35.4 | 35.2 |
| ADF | 19.5 | 19.6 | 19.0 | 18.6 |
| NFC^4^ | 37.1 | 33.1 | 37.9 | 34.9 |
| EE | 5.00 | 8.20 | 5.00 | 8.20 |
| Ca^5^ | 0.20 | 0.20 | 0.20 | 0.20 |
| P^5^ | 0.10 | 0.10 | 0.10 | 0.10 |
| Glucosinolates, mg/g | 0.34 | 0.36 | 1.18 | 2.27 |
| NEl^6^, Mcal/kg DM | 1.60 | 1.80 | 1.60 | 1.70 |

^1.^NCS5=non-camelina seed inclusion at 5% dietary EE; NCS8=non-camelina seed inclusion at 8% dietary EE; CS5=low camelina seed inclusion at 5% dietary EE; CS8=high camelina seed inclusion at 8% dietary EE.

^2^Megalac, Church & Dwight Co., Inc., Princeton, NJ.

^3^Provided (per kg of DM): 955 g of NaCl, 3,500 ppm of Zn, 2,000 ppm of Fe, 1,800 ppm of Mn, 280 ppm of Cu, 100 ppm of I, and 60 ppm of Co.

^4^estimated according to NRC (2001), using the following equation: NFC = 100 – (% NDF + % CP + % fat + % ash).

^5^Estimated according to NRC (2001).

^6^NEl = Net energy for lactation, estimated using the NRC (2001) model.

Supplementary Table *2*. Metadata of ruminal digesta from liquid- and Solid- fraction

|  |  |  |  |  |  | Normalized to 13,000 sequences per sample^1^ | | | |
| --- | --- | --- | --- | --- | --- | --- | --- | --- | --- |
| Groups | Fermenter No. | Periods | Fractions | Total Sequences reads | Good's Coverage (%) | Ace | Chao | Shannon | Inverse-Simpson |
| F1P2_L | 1 | 2 | Liquid | 30149 | 99.48 | 1140 | 1153 | 4.88 | 37.5 |
| F1P3_L | 1 | 3 | Liquid | 51325 | 99.87 | 1101 | 654.5 | 2.65 | 7.51 |
| F1P3_P | 1 | 3 | Solid | 59758 | 99.90 | 374.7 | 300.0 | 3.30 | 11.1 |
| F1P4_L | 1 | 4 | Liquid | 58866 | 99.75 | 624.7 | 645.1 | 3.31 | 8.39 |
| F1P4_P | 1 | 4 | Solid | 63483 | 99.90 | 449.3 | 372.6 | 3.20 | 9.24 |
| F1P5_L | 1 | 1 | Liquid | 36732 | 99.80 | 649.5 | 548.8 | 3.22 | 11.4 |
| F1P5_P | 1 | 1 | Solid | 124379 | 99.96 | 200.3 | 170.3 | 0.76 | 1.33 |
| F2P2_L | 2 | 2 | Liquid | 25023 | 99.24 | 2068 | 1529 | 3.84 | 10.5 |
| F2P2_P | 2 | 2 | Solid | 39098 | 99.57 | 3459 | 2077 | 4.28 | 19.0 |
| F2P3_L | 2 | 3 | Liquid | 29785 | 99.50 | 1308 | 1043 | 2.77 | 5.53 |
| F2P3_P | 2 | 3 | Solid | 32306 | 99.79 | 765.2 | 531.2 | 3.08 | 6.69 |
| F2P4_P | 2 | 4 | Solid | 66754 | 99.81 | 776.4 | 595.3 | 3.07 | 5.80 |
| F2P5_L | 2 | 1 | Liquid | 35815 | 99.78 | 974.1 | 763.4 | 2.23 | 3.82 |
| F2P5_P | 2 | 1 | Solid | 40477 | 99.76 | 1258 | 983.5 | 3.76 | 19.0 |
| F3P2_L | 3 | 2 | Liquid | 24939 | 99.57 | 1575 | 1006 | 2.84 | 4.26 |
| F3P2_P | 3 | 2 | Solid | 47564 | 99.77 | 3904 | 2309 | 3.84 | 12.7 |
| F3P3_L | 3 | 3 | Liquid | 14910 | 98.67 | 1018 | 958.6 | 3.88 | 7.96 |
| F3P3_P | 3 | 3 | Solid | 47113 | 99.56 | 4594 | 2981 | 4.67 | 14.6 |
| F3P4_L | 3 | 4 | Liquid | 34699 | 99.73 | 1627 | 1186 | 2.65 | 6.33 |
| F3P4_P | 3 | 4 | Solid | 85402 | 99.74 | 1587 | 1327 | 4.62 | 20.6 |
| F3P5_L | 3 | 1 | Liquid | 31321 | 99.65 | 1037 | 767.1 | 2.64 | 5.67 |
| F3P5_P | 3 | 1 | Solid | 44190 | 99.62 | 3746 | 2864 | 4.45 | 19.6 |
| F4P2_L | 4 | 2 | Liquid | 43431 | 99.72 | 2330 | 1697 | 2.88 | 6.80 |
| F4P2_P | 4 | 2 | Solid | 89198 | 99.87 | 577.4 | 470.6 | 2.71 | 4.86 |
| F4P3_L | 4 | 3 | Liquid | 43730 | 99.45 | 5125 | 3447 | 4.07 | 10.55 |
| F4P3_P | 4 | 3 | Solid | 59181 | 99.61 | 1586 | 1268 | 4.42 | 15.0 |
| F4P4_L | 4 | 4 | Liquid | 32942 | 99.50 | 1517 | 1162 | 3.52 | 6.13 |
| F4P4_P | 4 | 4 | Solid | 70239 | 99.76 | 1143 | 1011 | 3.39 | 5.50 |
| F4P5_L | 4 | 1 | Liquid | 27259 | 99.55 | 1247 | 884.0 | 3.28 | 9.96 |
| F4P5_P | 4 | 1 | Solid | 63790 | 99.83 | 1024 | 760.8 | 3.30 | 10.3 |
| F5P2_L | 5 | 2 | Liquid | 34390 | 99.66 | 983.3 | 728.1 | 2.77 | 4.62 |
| F5P2_P | 5 | 2 | Solid | 68388 | 99.82 | 802.7 | 647.1 | 3.16 | 7.88 |
| F5P3_L | 5 | 3 | Liquid | 27550 | 99.33 | 1517 | 1204 | 3.60 | 7.46 |
| F5P3_P | 5 | 3 | Solid | 69247 | 99.69 | 1399 | 1144 | 4.19 | 14.7 |
| F5P4_L | 5 | 4 | Liquid | 34374 | 99.60 | 1429 | 1005 | 2.72 | 3.70 |
| F5P4_P | 5 | 4 | Solid | 61364 | 99.72 | 1175 | 1038 | 3.80 | 8.39 |
| F5P5_L | 5 | 1 | Liquid | 22227 | 98.87 | 2692 | 1859 | 4.63 | 20.1 |
| F5P5_P | 5 | 1 | Solid | 60440 | 99.80 | 938.7 | 826.5 | 3.62 | 14.1 |
| F6P2_L | 6 | 2 | Liquid | 25418 | 99.06 | 2695 | 2036 | 5.24 | 33.9 |
| F6P2_P | 6 | 2 | Solid | 65630 | 99.62 | 1955 | 1571 | 4.79 | 33.2 |
| F6P3_L | 6 | 3 | Liquid | 51301 | 99.82 | 1987 | 1250 | 1.99 | 3.69 |
| F6P3_P | 6 | 3 | Solid | 66325 | 99.87 | 723.2 | 524.4 | 2.55 | 4.77 |
| F6P4_L | 6 | 4 | Liquid | 108972 | 99.73 | 1671 | 1505 | 4.39 | 15.2 |
| F6P4_P | 6 | 4 | Solid | 46068 | 99.77 | 2410 | 1506 | 2.99 | 5.43 |
| F6P5_L | 6 | 1 | Liquid | 91464 | 99.78 | 1302 | 1053 | 3.99 | 13.8 |
| F6P5_P | 6 | 1 | Solid | 40048 | 99.63 | 1836 | 1353 | 3.85 | 16.8 |
| F7P2_L | 7 | 2 | Liquid | 43688 | 99.47 | 4094 | 2902 | 4.90 | 35.3 |
| F7P2_P | 7 | 2 | Solid | 87183 | 99.75 | 1551 | 1299 | 4.48 | 21.9 |
| F7P3_L | 7 | 3 | Liquid | 71317 | 99.90 | 679.3 | 465.8 | 2.59 | 6.42 |
| F7P3_P | 7 | 3 | Solid | 104135 | 99.88 | 489.1 | 375.0 | 2.48 | 4.86 |
| F7P4_L | 7 | 4 | Liquid | 66402 | 99.82 | 836.4 | 684.0 | 2.84 | 5.23 |
| F7P4_P | 7 | 4 | Solid | 46121 | 99.70 | 2537 | 1699 | 3.09 | 4.39 |
| F7P5_L | 7 | 1 | Liquid | 47572 | 99.79 | 2647 | 1561 | 3.56 | 15.4 |
| F7P5_P | 7 | 1 | Solid | 58360 | 99.87 | 517.5 | 383.6 | 2.04 | 2.74 |
| F8P2_L | 8 | 2 | Liquid | 57288 | 99.80 | 1006 | 760.0 | 2.33 | 3.69 |
| F8P2_P | 8 | 2 | Solid | 66955 | 99.79 | 1094 | 910.0 | 3.36 | 7.91 |
| F8P3_L | 8 | 3 | Liquid | 45392 | 99.38 | 4166 | 3183 | 4.58 | 8.77 |
| F8P3_P | 8 | 3 | Solid | 58724 | 99.58 | 1629 | 1248 | 4.42 | 14.1 |
| F8P4_L | 8 | 4 | Liquid | 73629 | 99.89 | 931.2 | 637.2 | 2.72 | 5.58 |
| F8P4_P | 8 | 4 | Solid | 48333 | 99.65 | 5996 | 3301 | 4.26 | 20.5 |
| F8P5_L | 8 | 1 | Liquid | 71190 | 99.92 | 413.5 | 292.4 | 1.63 | 2.57 |
| F8P5_P | 8 | 1 | Solid | 40464 | 99.75 | 2789 | 1743 | 4.03 | 21.3 |

1 ACE and Chao, the richness index; Shannon and inverse-Simpson, the diversity index; 13,000, were selected based on the smallest sequence reads of the groups.

Supplementary Table 4. P-values associated with PERMANOVA

|  |  |  | Index | |
| --- | --- | --- | --- | --- |
| Items^1^ | **Fractions** | **Test** | **Bray-Curtis** | **Jaccard** |
| CS | Liquid | PERMANOVA | <0.01 | <0.01 |
|  | Solid | PERMANOVA | <0.01 | <0.01 |
| EE | Liquid | PERMANOVA | 0.53 | 0.43 |
|  | Solid | PERMANOVA | 0.82 | 0.78 |
| CS X EE | Liquid | PERMANOVA | 0.43 | 0.45 |
|  | Solid | PERMANOVA | 0.58 | 0.7 |
| Fractions |  | PERMANOVA | <0.01 | <0.01 |

1 CS=effects of camelina seed; EE= effects of dietary EE levels; CS X EE=interaction CS and EE.

Supplementary Table 5. Effect of camelina seed supplementation and dietary EE levels on the relative abundance of ruminal bacterial families in both of liquid and solid fractions^1^

| **Items** | **Fractions** | **Treatments^2^** | | | | **SEM** | **P-Value^3^** | | |
| --- | --- | --- | --- | --- | --- | --- | --- | --- | --- |
|  |  | **NCS5** | **NCS8** | **CS5** | **CS8** |  | **CS** | **EE** | **CS x EE** |
| *Lachnospiraceae* | Solid | 28.3 | 23 | 17.9 | 15.9 | 2.85 | <0.01 | 0.22 | 0.57 |
| *Erysipelotrichaceae* | Solid | 1.86 | 3.14 | 14.5 | 10.6 | 2.65 | <0.01 | 0.64 | 0.35 |
| *Ruminococcaceae* | Solid | 3.21 | 3.01 | 2.1 | 0.38 | 0.61 | 0.03 | 0.43 | 0.66 |
| *Paraprevotellaceae* | Solid | 4.87 | 2.81 | 2.33 | 2.69 | 0.73 | 0.04 | 0.18 | 0.06 |
| *Spirochaetaceae* | Solid | 0.33 | 0.25 | 0.14 | 0.09 | 0.06 | 0.01 | 0.29 | 0.84 |
| *Fibrobacteraceae* | Solid | 0.61 | 0.78 | 0.43 | 0.31 | 0.16 | 0.05 | 0.88 | 0.37 |
| *F16* | Solid | 0.44 | 0.43 | 0.08 | 0.11 | 0.12 | <0.01 | 0.95 | 0.89 |
| *Bifidobacteriaceae* | Solid | 0.01 | 0.01 | 0.02 | 0.02 | 0.01 | 0.04 | 0.98 | 0.98 |
| Others | Solid | 0.33 | 0.54 | 0.33 | 0.34 | 0.22 | <0.01 | 0.33 | 0.34 |
| *Prevotellaceae* | Liquid | 33.7 | 30.6 | 42.2 | 30.6 | 5.13 | 0.15 | 0.02 | 0.15 |
| *Lachnospiraceae* | Liquid | 9.60 | 12.4 | 6.95 | 5.02 | 1.73 | <0.01 | 0.71 | 0.06 |
| *Erysipelotrichaceae* | Liquid | 8.09 | 5.20 | 18.3 | 24 | 5.77 | <0.01 | 0.71 | 0.26 |
| *Succinivibrionaceae* | Liquid | 6.83 | 1.83 | 9.84 | 8.68 | 2.23 | 0.01 | 0.1 | 0.29 |
| *Veillonellaceae* | Liquid | 4.04 | 3.45 | 6.92 | 7.73 | 1.48 | <0.01 | 0.86 | 0.3 |
| *Ruminococcaceae* | Liquid | 2.75 | 3.24 | 1.14 | 0.63 | 0.62 | <0.01 | 0.99 | 0.33 |
| *Paraprevotellaceae* | Liquid | 2.04 | 3.24 | 1.39 | 1.56 | 0.57 | 0.02 | 0.14 | 0.26 |
| *Clostridiaceae* | Liquid | 1.70 | 3.02 | 0.78 | 0.35 | 1.00 | <0.01 | 0.4 | 0.11 |
| *Spirochaetaceae* | Liquid | 1.63 | 1.07 | 0.67 | 0.35 | 0.38 | 0.01 | 0.16 | 0.69 |
| *Fibrobacteraceae* | Liquid | 1.06 | 1.21 | 0.64 | 0.24 | 0.24 | <0.01 | 0.59 | 0.25 |
| *Bacteroidaceae* | Liquid | 0.99 | 1.43 | 0.16 | 0.46 | 0.28 | <0.01 | 0.14 | 0.8 |
| *Mogibacteriaceae* | Liquid | 0.44 | 0.71 | 0.32 | 0.27 | 0.12 | <0.01 | 0.11 | 0.02 |
| *S24_7* | Liquid | 0.35 | 0.39 | 0.53 | 1.12 | 0.25 | <0.01 | 0.05 | 0.07 |
| *Anaeroplasmataceae* | Liquid | 0.21 | 0.42 | 0.16 | 0.03 | 0.07 | <0.01 | 0.48 | <0.01 |
| *F16* | Liquid | 0.15 | 0.22 | 0.06 | 0.01 | 0.04 | <0.01 | 0.9 | 0.15 |
| *RFP12* | Liquid | 0.14 | 0.23 | 0.04 | 0.06 | 0.07 | <0.01 | 0.18 | 0.38 |
| *Desulfovibrionaceae* | Liquid | 0.12 | 0.17 | 0.09 | 0.08 | 0.02 | <0.01 | 0.18 | 0.03 |
| unclassified | Liquid | 15.8 | 24.4 | 6.11 | 13.8 | 6.18 | 0.02 | 0.07 | 0.91 |
| Others | Liquid | 0.96 | 1.31 | 0.33 | 0.34 | 0.22 | <0.01 | 0.33 | 0.34 |

^1^Only the relative abundance of families higher than 0.1% and significantly affected by treatments were presented.

^2^NCS5= non-camelina seed inclusion at 5% dietary EE; NCS8= non-camelina seed inclusion at 8% dietary EE; CS5= low camelina seed inclusion at 5% dietary EE; CS8= high camelina seed inclusion at 8% dietary EE.

^3^CS=effects of camelina seed; EE= effects of dietary EE levels; CC x EE=the interaction between CC and EE.

Supplementary Table 6. Effect of camelina seed supplementation and dietary EE levels on the relative abundance of ruminal bacterial genera in effluent digesta1

| Items | Fractions | Treatments^2^ | | | | SEM | P-Value^3^ | | |
| --- | --- | --- | --- | --- | --- | --- | --- | --- | --- |
|  |  | **NCS5** | **NCS8** | **CS5** | **CS8** |  | **CS** | **EE** | **CS x EE** |
| *Butyrivibrio* | Solid | 6.6 | 7.14 | 5.52 | 3.2 | 1.42 | 0.03 | 0.42 | 0.2 |
| *Ruminococcus* | Solid | 2.41 | 2.2 | 1.5 | 0.83 | 0.5 | 0.03 | 0.4 | 0.66 |
| *Treponema* | Solid | 0.32 | 0.24 | 0.13 | 0.09 | 0.06 | <0.01 | 0.31 | 0.81 |
| *Fibrobacter* | Solid | 0.61 | 0.78 | 0.43 | 0.31 | 0.16 | 0.05 | 0.88 | 0.37 |
| *Oscillospira* | solid | 0.27 | 0.3 | 0.2 | 0.15 | 0.04 | 0.02 | 0.75 | 0.39 |
| *Selenomonas* | Solid | 0.08 | 0.08 | 0.14 | 0.13 | 0.04 | 0.02 | 0.58 | 0.77 |
| *Anaerovibrio* | Solid | 0.35 | 0.28 | 0.89 | 0.6 | 0.33 | <0.01 | 0.23 | 0.45 |
| *Bifidobacterium* | Solid | 0.01 | 0.01 | 0.02 | 0.02 | 0.01 | 0.04 | 0.98 | 0.98 |
| *Prevotella* | Liquid | 33.7 | 30.6 | 42.2 | 30.6 | 4.98 | 0.15 | 0.02 | 0.16 |
| *Succinivibrio* | Liquid | 6.56 | 1.56 | 9.22 | 7.2 | 2.32 | 0.02 | 0.04 | 0.36 |
| *Butyrivibrio* | Liquid | 2.47 | 4.05 | 1.43 | 0.67 | 0.57 | <0.01 | 0.36 | 0.02 |
| *Megasphaera* | Liquid | 2.07 | 1.46 | 4.98 | 5.01 | 1.8 | <0.01 | 0.66 | 0.63 |
| *Ruminococcus* | Liquid | 1.96 | 2.3 | 0.75 | 0.32 | 0.45 | <0.01 | 0.91 | 0.31 |
| *Clostridium* | Liquid | 1.92 | 3.37 | 1.04 | 0.92 | 1.01 | <0.01 | 0.22 | 0.15 |
| *Treponema* | Liquid | 1.6 | 1.05 | 0.62 | 0.34 | 0.37 | <0.01 | 0.17 | 0.65 |
| *YRC22* | Liquid | 1.54 | 2.64 | 1.07 | 1.25 | 0.56 | 0.04 | 0.16 | 0.31 |
| *Fibrobacter* | Liquid | 1.06 | 1.21 | 0.64 | 0.24 | 0.24 | <0.01 | 0.59 | 0.25 |
| *Coprococcus* | Liquid | 0.9 | 1.04 | 0.38 | 0.42 | 0.18 | <0.01 | 0.62 | 0.77 |
| *BF311* | Liquid | 0.51 | 1.15 | 0.11 | 0.01 | 0.21 | <0.01 | 0.18 | 0.07 |
| *CF231* | Liquid | 0.43 | 0.53 | 0.32 | 0.3 | 0.11 | 0.02 | 0.57 | 0.38 |
| *Oscillospira* | Liquid | 0.26 | 0.32 | 0.18 | 0.11 | 0.06 | <0.01 | 0.86 | 0.14 |
| *Anaeroplasma* | Liquid | 0.2 | 0.42 | 0.16 | 0.03 | 0.07 | <0.01 | 0.47 | 0.03 |
| *Desulfovibrio* | Liquid | 0.12 | 0.17 | 0.09 | 0.08 | 0.02 | <0.01 | 0.19 | 0.03 |
| *Adlercreutzia* | Liquid | 0.12 | 0.19 | 0.07 | 0.06 | 0.02 | <0.01 | 0.27 | 0.11 |
| *Selenomonas* | Liquid | 0.11 | 0.13 | 0.27 | 0.36 | 0.09 | 0.03 | 0.54 | 0.75 |
| *Anaerovibrio* | Liquid | 0.09 | 0.11 | 0.23 | 0.43 | 0.15 | 0.02 | 0.22 | 0.33 |
| unclassified | Liquid | 28.9 | 37.3 | 28.7 | 39.4 | 7.31 | 0.83 | 0.03 | 0.78 |

^1^Only the relative abundance of families higher than 0.1% and significantly affected by treatments were presented.

^2^NCS5= non-camelina seed inclusion at 5% dietary EE; NCS8= non-camelina seed inclusion at 8% dietary EE; CS5= low camelina seed inclusion at 5% dietary EE; CS8= high camelina seed inclusion at 8% dietary EE.

^3^CS=effects of camelina seed; EE= effects of dietary EE levels; CC x EE=the interaction between CC and EE.

**Supplementary Text 1.** Sequence data processing code in mothur:

make.contigs(file=XD.txt,processors=10)

summary.seqs(fasta=XD.trim.contigs.fasta)

screen.seqs(fasta=XD.trim.contigs.fasta,group=XD.contigs.groups,, maxambig=0, maxhomop=8, minlength=200, maxlength=500)

summary.seqs(fasta=XD.trim.contigs.good.fasta)

unique.seqs(fasta=XD.trim.contigs.good.fasta)

count.seqs(name=XD.trim.contigs.good.names,group=XD.contigs.good.groups)

summary.seqs(fasta=XD.trim.contigs.good.unique.fasta,count=XD.trim.contigs.good.count_table)

align.seqs(fasta=XD.trim.contigs.good.unique.fasta, reference= silva.nr_v123.align, flip=T)

summary.seqs(fasta=XD.trim.contigs.good.unique.align,count=XD.trim.contigs.good.count_table)

screen.seqs(fasta=XD.trim.contigs.good.unique.align,count=XD.trim.contigs.good.count_table, summary=XD.trim.contigs.good.unique.summary, start=13862, end=23444)

summary.seqs(fasta=XD.trim.contigs.good.unique.good.align, count=XD.trim.contigs.good.good.count_table)

filter.seqs(fasta=XD.trim.contigs.good.unique.good.align, vertical=T, trump=.)

unique.seqs(fasta=XD.trim.contigs.good.unique.good.filter.fasta, count=XD.trim.contigs.good.good.count_table)

pre.cluster(fasta=XD.trim.contigs.good.unique.good.filter.unique.fasta,count=XD.trim.contigs.good.unique.good.filter.count_table, diffs=2)

summary.seqs(fasta=XD.trim.contigs.good.unique.good.filter.unique.precluster.fasta, count=XD.trim.contigs.good.unique.good.filter.unique.precluster.count_table)

chimera.uchime(fasta=XD.trim.contigs.good.unique.good.filter.unique.precluster.fasta, count=XD.trim.contigs.good.unique.good.filter.unique.precluster.count_table)

remove.seqs(fasta=XD.trim.contigs.good.unique.good.filter.unique.precluster.fasta, count=XD.trim.contigs.good.unique.good.filter.unique.precluster.count_table, accnos=XD.trim.contigs.good.unique.good.filter.unique.precluster.denovo.uchime.accnos)

summary.seqs(fasta=XD.trim.contigs.good.unique.good.filter.unique.precluster.pick.fasta, count=XD.trim.contigs.good.unique.good.filter.unique.precluster.pick.count_table)

classify.seqs(fasta=XD.trim.contigs.good.unique.good.filter.unique.precluster.pick.fasta, count=XD.trim.contigs.good.unique.good.filter.unique.precluster.uchime.pick.count_table,reference= silva.nr_v123.align, taxonomy= silva.nr_v123.tax, cutoff=60)

remove.lineage(fasta=XD.trim.contigs.good.unique.good.filter.unique.precluster.pick.fasta, count=XD.trim.contigs.good.unique.good.filter.unique.precluster.pick.count_table, taxonomy=XD.trim.contigs.good.unique.good.filter.unique.precluster.pick.nr_v123.wang.taxonomy,taxon=unknown;-Archaea;-Eukaryota;-Bacteria;Cyanobacteria;-Bacteria;Proteobacteria;Alphaproteobacteria;Rickettsiales;mitochondria;)

summary.seqs(fasta=XD.trim.contigs.good.unique.good.filter.unique.precluster.pick.pick.fasta, count=XD.trim.contigs.good.unique.good.filter.unique.precluster.pick.pick.count_table)

count.groups(count=XD.trim.contigs.good.unique.good.filter.unique.precluster.pick.pick.count_table)

split.abund(fasta=XD.trim.contigs.good.unique.good.filter.unique.precluster.pick.pick.fasta, count=XD.trim.contigs.good.unique.good.filter.unique.precluster.pick.pick.count_table, cutoff=1)

count.groups(count=XD.trim.contigs.good.unique.good.filter.unique.precluster.pick.pick.abund.count_table)

summary.seqs(fasta=XD.trim.contigs.good.unique.good.filter.unique.precluster.pick.pick.abund.fasta, count=XD.trim.contigs.good.unique.good.filter.unique.precluster.pick.pick.abund.count_table)

system(cp XD.trim.contigs.good.unique.good.filter.unique.precluster.pick.pick.abund.fasta XD.final.fasta)

system (cp XD.trim.contigs.good.unique.good.filter.unique.precluster.pick.pick.abund.count_table XD.final.count_table)

dist.seqs(fasta=XD.final.fasta)

cluster.split(column=XD.final.dist, count=XD.final.count_table, method=average, cutoff=0.1)

make.shared(list=XD.final.an.unique_list.list, count=XD.final.count_table, label=0.03)

normalize.shared(shared=XD.final.an.unique_list.shared, norm=13000)

summary.single(shared=XD.final.an.unique_list.0.03.norm.shared, label=0.03, calc=nseqs-sobs-coverage-ace-chao-shannon-simpson-invsimpson)

classify.seqs(fasta=XD.final.fasta,count=XD.final.count_table, template=gg_13_8_99.fasta,taxonomy=gg_13_8_99.gg.tax, cutoff=80)

classify.otu(list=XD.final.an.unique_list.list,taxonomy=XD.final.gg.wang.taxonomy,count=XD.final.count_table,label=0.03, cutoff=80, basis=otu, probs=F)

get.oturep(column=XD.final.dist,list=XD.final.an.unique_list.list,count=XD.final.count_table,fasta=XD.final.fasta,label=0.03, large=true)
